# Retraction: Inhibition of YAP function overcomes BRAF inhibitor resistance in melanoma cancer stem cells

**DOI:** 10.18632/oncotarget.28792

**Published:** 2025-12-31

**Authors:** Matthew L. Fisher, Daniel Grun, Gautam Adhikary, Wen Xu, Richard L. Eckert

**Affiliations:** ^1^Department of Biochemistry and Molecular Biology, University of Maryland School of Medicine, Baltimore, Maryland, 21201, USA; ^2^Department of Dermatology, University of Maryland School of Medicine, Baltimore, Maryland, 21201, USA; ^3^Department of Reproductive Biology, University of Maryland School of Medicine, Baltimore, Maryland, 21201, USA; ^4^The Marlene and Stewart Greenebaum Comprehensive Cancer Center, University of Maryland School of Medicine, Baltimore, Maryland, 21201, USA

The initial request from the authors was for a correction, citing a mistaken placement of images and providing original blots. However, the Scientific Integrity Office conducted an independent verification of the results and discovered additional instances of image manipulation:

1. Duplication of Western blots in Figure 4G and 4H (b-actin in A375-PLX-R cells).
2. Reuse of the YAP-1 blot from Figure 1F in Figure 3A, illustrating a different experiment.

In light of the number of necessary corrections and in agreement with the recommendations from the ORI (NIH), the Editorial decision has been made to retract this paper.

Original article: Oncotarget. 2017; 8:110257–110272. 110257-110272. https://doi.org/10.18632/oncotarget.22628

